# The acute wheezy adult with airways disease in the emergency department: a retrospective case-note review of exacerbations of COPD

**DOI:** 10.2147/COPD.S190085

**Published:** 2019-05-07

**Authors:** REK Russell, S Beer, ID Pavord, R Pullinger, M Bafadhel

**Affiliations:** 1 Respiratory Medicine Unit, Nuffield Department of Clinical Medicine, University of Oxford, Oxford, UK; 2 NIHR Oxford Biomedical Research Centre, University of Oxford, Oxford, UK; 3 Department of Emergency Medicine, Oxford University Hospitals NHS Foundation Trust, Oxford, UK

**Keywords:** COPD, exacerbation, eosinophils, mortality, predictors

## Abstract

**Introduction:** There has been an increase in interest in the peripheral blood eosinophil count as a biomarker in COPD. Few studies have examined the eosinophil count in patients attending the emergency department (ED) with acute exacerbations of COPD (AECOPD). We investigated the relationship between the blood eosinophil and other variables collected routinely at ED presentation and outcomes.

**Methods:** Retrospective case note review of patients attending the ED with an AECOPD over 18 months. Demographic, clinical and pharmacological data were analyzed at the time of presentation, and clinical outcomes relating to hospital admission, length of hospital stay and mortality were investigated.

**Results:** There were 743 AECOPD index events in 537 patients. Over half (57%) of all attendees were admitted to hospital. They were older, reported an increased number of exacerbations and higher levels of total leukocytes and neutrophils. Length of stay was shorter in patients with a blood eosinophil count ≥2% compared to <2% (median (IQR) 3 days (1–7) vs 4 days (2–8) respectively, *p*<0.05). Length of stay correlated with peripheral blood neutrophils (*r*=0.12, *p*=0.021), peripheral blood absolute and relative eosinophils (*r*=−0.12, *p*=0.024 and *r*=−0.11, *p*=0.035, respectively) and CRP (*r*=0.16, *p*=0.027). Non-eosinophilic AECOPD were associated with an increased risk of mortality during an exacerbation (*χ*^2^ 5.9, OR 3.08, 95% CI 1.19–7.96, *p*=0.015).

**Conclusion:** In exacerbations of COPD presenting to ED, a higher blood eosinophil count is associated with a shorter length of stay and reduced mortality.

## Introduction

COPD is common worldwide and associated with significant morbidity and mortality.^1^,^2^ Stable COPD is punctuated by periods of worsening of symptoms, termed exacerbations, akin to a significant lung attack. Acute exacerbations account for 15% of all UK emergency hospital admissions, 1 million hospital bed days, an expenditure of approximately £500 million and 130,000 hospitalizations per annum.^3^ In patients with an exacerbation of COPD that seek medical assistance in the emergency department (ED), over 50% of attendances are then hospitalized.^4^–^7^ The mainstays of treatment of acute exacerbations of COPD (AECOPD) are systemic corticosteroids and antibiotics.^3^,^8^ Current treatment strategies are associated with a significant risk of harm,^9^,^10^ and treatment failure resulting in hospital admission and re-treatment is common.^5^,^6^,^11^ There is an urgent requirement for improved strategies to target treatment to the patients with the best chance of net treatment response.

There is now compelling evidence that higher sputum or peripheral blood eosinophil counts in patients with airway disease are associated with an increased likelihood of response to corticosteroid therapy.^11^–^15^ Furthermore, low levels of eosinophils are associated with worsened outcomes in severe AECOPD.^16^ A single center intervention study in non-hospitalized acute exacerbations showed that blood eosinophil directed corticosteroid treatment resulted in fewer treatment failures, and a faster rate of symptomatic recovery.^11^ However, whether the peripheral blood eosinophil count in AECOPD in the ED can identify an “at risk” population remains unknown. In this retrospective case note review, we investigated the relationship between the peripheral blood eosinophil count and other variables collected routinely at ED presentation and outcomes in AECOPD attendances.

The primary objective of the study was to use routinely collected data at presentation to the ED to see if any were predictive of outcome. All analyses were driven by the data collected which included basic patient demographics, length of hospital stay, initial blood results. Data on treatment, either pre- or post-initial assessment was not collected.

## Methods

Retrospective and anonymized data for all attendances for an AECOPD were collected from the John Radcliffe Hospital, part of the Oxford University Hospitals Foundation NHS Trust from the 1st of February 2014 to the 31st of July 2015. The John Radcliffe Hospital houses a large ED, providing direct health coverage to a population of 650,000 people. AECOPD was defined according to Anthonisen criteria^17^ and health-care utilization^18^ and hospital admission were defined as direct admission to an inpatient hospital bed, high-dependency unit or intensive care ward, following the initiation of treatment in the ED. Data from patients, with an ICD-10 primary code of Acute Exacerbation of COPD (J44.1), were collated. Patients with any diagnosis other than AECOPD were excluded from analysis. Data collection included demography, vital status, time of attendance at the ED, time spent in the ED, length of hospital stay following admission and treatment. The biological inflammatory state was assessed using the CRP and the full blood count, which included the total peripheral blood leukocyte and the total differential neutrophil and eosinophil count. Participants with missing information regarding admission status, treatment or biological status were excluded. This study was a retrospective review of data performed as a service review and as such did not require ethics review.

The eosinophilic exacerbation phenotype was defined as a peripheral blood eosinophil count on admission was ≥2% of the total leukocyte count in initial testing in ED. Repeated events were defined as being always eosinophilic (all captured AECOPD events), mostly eosinophilic (>50% of events), rarely eosinophilic (<50% of events) and never eosinophilic (none of the captured AECOPD events). These definitions were specified before the data were collected and before analysis was performed.

### Statistical analysis

All data were analyzed using Version 7 Prism (GraphPad, San Diego, CA) and SPSS 22 for Windows (SPSS Inc. Chicago, IL) and presented using column statistics. Parametric and non-parametric data are presented as mean (SEM) and median (IQR), respectively unless stated. Log transformation of cell counts was performed and is presented as geometric mean (95%CI of the geometric mean). The Student *t*-test and the Mann–Whitney *U*-test were used to analyze parametric and non-parametric continuous variables, respectively. For comparison of three or more groups, the one-way analysis of variance or Kruskal–Wallis test was used for parametric and non-parametric data. The Fishers Exact test and the Chi-squared test werre used to compare categorical data, and the Student’s *t*-test and log-rank Mantel Cox test were used to compare the length of stay. Logistic regression analysis was performed to determine predictors of hospital admission and mortality (inclusion and exclusion criteria were based on a*p* -value of <0.05). A*p* -value of <0.05 was taken as the threshold of significance for all statistical testing.

## Results

**Figure 1:**
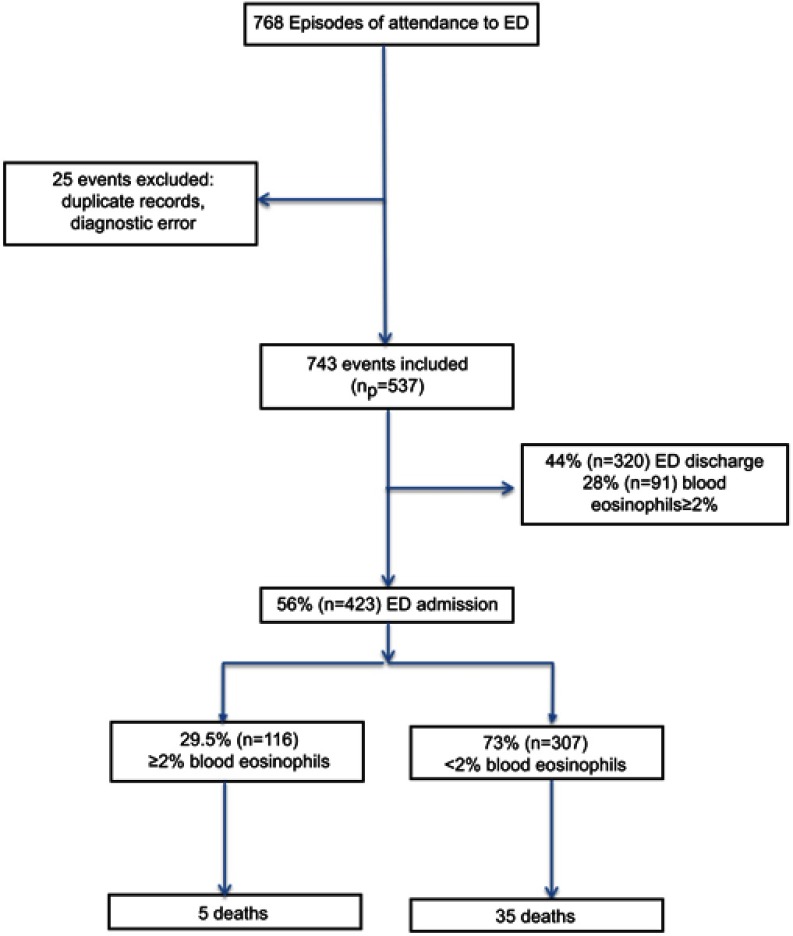
Study consort diagram. **Abbreviation:** ED, emergency department.

During the study period, a total of 768 episodes of AECOPD were seen in ED (Figure 1). After exclusion of missing data, there were 743 episodes from 537 patients (52% men, n=280) with a mean age (range) of 71 years (30–101). The mean (SD) ED exacerbation rate in the population over the study duration was 1.4 (1.0), with no differences in ED exacerbation frequency between men and women. At the index ED event, 29.5% had a blood eosinophil count of ≥2%. The geometric mean (95%CI) neutrophil and eosinophil count was 8.6×10^9^ cells/L (8.3–8.9) and 0.06×10^9^ cells/L (0.05–0.07), respectively. The median (IQR) CRP was 19.4 g/L (5.8–58.5).

### Hospital admission

Of all of the AECOPD seen in the ED, 57% (423 events) were admitted to hospital. Patients admitted to hospital were older (mean difference 2.4 years, 95%CI 0.9–4.0, *p*=0.002) and had a statistically higher level of prior ED attendance for AECOPD (mean difference 0.2, 95%CI 0.03–0.36, *p*=0.018). The blood profiles of the patients admitted and discharged from ED are presented in Table 1. Logistic regression analysis determined that higher total neutrophils and lower % eosinophils were independent predictors of admission of AECOPD from ED (Table 2).

**Table 1:** Biochemical profiles at the index event in hospitalized and discharged from ED

	Discharged	Admitted	*p*-Value
Total leukocytes^¥^ x10^9^cells/L^¥^	10.8 (10.3–11.3)	11.8 (11.4–12.3)	0.005
Total neutrophils^¥^ x10^9^cells/L^¥^	8.0 (7.5–8.5)	9.1 (8.7–9.5)	0.001
Total eosinophils^¥^ x10^9^cells/L^¥^	0.06 (0.05–0.08)	0.05 (0.05–0.06)	0.142
% eosinophil count*	1.7 (1.4–2.0)	1.6 (1.4–1.9)	0.625
Total CRP^§^ mg/L^§^	17.5 (3.6–56.0)	23.4 (7.1–59.6)	0.102

**Notes:** *Mean (95% CI); ^¥^Geometric mean (95%CI); ^§^Median (interquartile range); ^δ^Student *t*-test or Mann–Whitney.

**Abbreviation: **ED, emergency department.

**Table 2:** Blood test predictors of hospitalization of ED AECOPD

	B	S.E.	Wald	df	Sig.
Total leukocytes	−4.519	2.419	3.491	1	0.062
Total neutrophils	4.947	2.071	5.706	1	0.017
Total eosinophils	−0.359	0.265	1.832	1	0.176
% eosinophil count	0.172	0.076	5.070	1	0.024
Total CRP	−0.001	0.002	0.291	1	0.590
Constant	−0.124	1.032	0.014	1	0.904

**Note:** Nagelkerke *R*^2^=0.046.

**Abbreviations:** AECOPD, acute exacerbations of COPD; ED, emergency department; B, beta co-efficient ; S.E., standard error; Wald, Wald statistic; df, degrees of freedom; Sig., statistical significance.

### Length of stay

**Figure 2:**
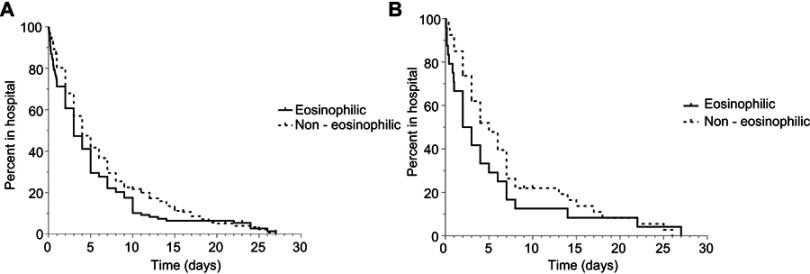
(**A**) Length of stay Kaplan–Meier curves according to inflammatory phenotype. (**B**). Length of stay Kaplan–Meier curves according to inflammatory phenotype treated with systemic corticosteroids.

Length of stay was significantly shorter in eosinophilic compared to non-eosinophilic acute exacerbations (by 1 day (median (IQR) 4 days (2–8) vs 3 days (1–7), *p*=0.048; Figure 2A). Length of hospital stay correlated negatively with peripheral blood absolute and relative eosinophils (*r*=−0.12, *p*=0.024 and *r*=−0.11, *p*=0.035, respectively) and positively with peripheral blood neutrophils (*r*=0.12, *p*=0.021) and CRP (*r*=0.16, *p*=0.027). Data on the prior use of systemic corticosteroid prescription were available for 162 events. For those receiving their first systemic corticosteroid prescription in the ED, the median (IQR) length of stay was 2.5 (0.9–6.6) days in eosinophilic acute exacerbations compared to 5.0 (2.0–7.3) days in non-eosinophilic acute exacerbations (Mantel-Cox Chi-squared 1.519, HR 1.33 (95%CI 0.85–2.40), *p*=0.218, Figure 2B).

### Repeat exacerbation events

One hundred and fifteen patients had repeated ED acute exacerbations with a mean (SD) number of events of 2.8 (1.4). The majority of patients (84.4%) presented to the ED with AECOPD only once, with 10.5% presenting twice to ED during the study period. Analysis of the blood profiles of those with repeated events were compared with those in whom there was only a single event, demostrated no significant differences between these two groups. In repeat ED AECOPD events, almost 40% were associated with an eosinophilic acute exacerbation more than 50% of the time; whilst 48.1% never had a raised eosinophil count at acute exacerbation (defined as relative eosinophil count ≥2%) measured on each event. The total leukocytes, neutrophils and eosinophils were significantly different between the eosinophil exacerbation phenotypes (Table 3).

**Table 3:** Clinical and biochemical data from according to eosinophilic COPD exacerbation phenotype

	Always N=20	Frequently N=22	Rarely N=14	Never N=52	*p*-Value ^δ^
Male, n (%)	13 (65)	12 (55)	8 (57)	27 (52)	
Age* (years)	74 (52–88)	75 (64–86)	68 (50–80)	71 (38–99)	0.137
Exacerbation rate^Δ^	2.5 (1.1)	3.0 (1.7)	4.4 (1.7)	2.5 (1.0)	0.001
Eosinophilic exacerbations, %	100	54 (50–83)	27 (14–33)	0	<0.001
Total leukocytes^¥^ x10^9^cells/L	10.2 (8.8–11.8)	9.4 (8.3–10.8)	10.0 (8.3–12.1)	12.2 (10.7–13.9)	0.044
Total neutrophils^¥^ x10^9^cells/L	6.9 (5.7–8.4)	6.8 (5.8–8.0)	7.4 (5.8–9.4)	10.0 (8.7–11.5)	0.001
Total eosinophils^¥^ x10^9^cells/L	0.40 (0.22–0.71)	0.11 (0.05–0.21)	0.06 (0.02–0.13)	0.02 (0.02–0.04)	<0.001
Total CRP^§^ mg/L	7.9 (3.6–56.5)	5.7 (2.6–39.2)	17.7 (2.1–23.6)	21.9 (5.3–69.7)	0.294

**Notes:** *Mean (range); ^Δ^Mean (SD); ^¥^Geometric mean (95%CI); ^§^Median (interquartile range); ^δ^ANOVA, or Kruskal–Wallis.

### Mortality

There were 40 deaths during the period of study. There were 35 deaths in the non-eosinophilic acute exacerbations compared with 5 deaths in those with eosinophilic acute exacerbations (*χ*^2^ 5.9, OR 3.08, 95% CI 1.19–7.96, *p*=0.015). The peripheral blood eosinophil count was significantly lower at the index exacerbation in patients that died. Mortality was also associated with increasing age and an increase in blood total leukocyte count and neutrophil count measured at a presentation in ED (Table 4).

**Table 4:** Clinical and biochemical data from according to survival

	Survived (n=703)	Died (n=40)	*p*-Value
Male, n (%)	370 (53)	25 (63)	0.224
Age*	71 (30–100)	76 (39–101)	0.003
Total leukocytes^¥^ x10^9^cells/L	11;2 (10.9–11.6)	13.9 (11.9–16.2)	0.002
Total neutrophils^¥^ x10^9^cells/L	8.5 (8.2–8.8)	11.2 (9.4–13.4)	0.001
Total eosinophils^¥^ x10^9^cells/L	0.06 (0.05–0.07)	0.03 (0.02–0.04)	0.001
Total CRP^§^ mg/L	18.9 (5.6–58.8)	31.1 (15.3–55.4)	0.193

Notes: *Mean (range); ^¥^Geometric mean (95%CI); ^§^Median (interquartile range).

## Discussion

This is one of the largest cross-sectional studies of patients with AECOPD and the first to study those in the clinical setting of the ED. We have shown that routinely collected biomarkers at the time of admission relate differently to key outcomes, and that in spite of all events being labeled and managed in a similar manner, in reality, the events vary between individual patients and also sometimes from event to event in a single individual. Acute exacerbation events are thus heterogeneous. Our data, although not as detailed as previous cluster analysis of AECOPD in a community and hospital setting, describe phenotypes of AECOPD which is pragmatic and is predictive of outcome.^11^,^19^

In our study, higher blood eosinophil counts were associated with a shorter length of stay and greatly reduced mortality. Moreover, the finding that the use of oral corticosteroids in those with a raised eosinophil count, but not in those with a low eosinophil count, leads to a shorter length of stay suggests that it may be possible to use the blood eosinophil as a guide to who should receive oral corticosteroids. This finding is based upon this retrospective data and so needs to be interpreted with some caution. So far, only one randomized clinical trial has shown that the eosinophil count can be used to direct oral corticosteroids at the time of an exacerbation.^11^ In a small case-control (20 patients) study of in-patients with severe AECOPD, it was also shown that blood eosinophils ≥2% is associated with a shorter length of stay (9 days vs 11 days) and reduced total corticosteroid intake suggesting a better treatment response.^20^ In a French study of 167 patients admitted to hospital with AECOPD, 33% were found to have blood eosinophils >2% and eosinophilia and/or ≥ 200 eosinophils/µL was associated with an increased risk of 12-month COPD-related readmission (OR, 3.59 [95% CI, 1.65–7.82]; *p*=0.0013).^21^ A further study of a large number (1,704 patients) admitted to a tertiary center for AECOPD demonstrated that 20% had blood eosinophils >2% which was associated with a reduced length of stay (6.6 vs 7 days); whilst Six-month mortality rates were similar between the eosinophilic and the non-eosinophilic patients.^22^ These data vary due to the clinical setting, national treatment variance, enrolment criteria and in severity of disease and exacerbation, but their findings are consistent and in agreement with our data. There is no doubt that there will be significant advances in this area very soon as new studies report.

Previous studies have demonstrated that low levels of blood eosinophils are a poor prognostic sign potentially reflecting underlying infection (DECAF).^16^ In support of this, we found that total neutrophil count and CRP were elevated in those who died. It is also possible that higher blood eosinophil counts are associated with better outcomes because of an increased likelihood of response to steroid treatment. A recent meta-analysis of prednisolone treatment of AECOPD provides strong support for this interpretation, showing that oral prednisolone reduces the treatment failure rate six-fold in patients with a baseline blood eosinophil count >2%.^15^ It may be hypothesized that the increased rate of re-exacerbation in these patients may be due to an inadequate treatment course or a lack of maintenance treatment.

We found that patients were more likely to be discharged if they had a slightly lower neutrophil count. Whilst this was statistically significant (*p*=0.001) we do not think that this difference was clinically relevant. We were unable to demonstrate that CRP levels were predictive of admission or discharge from the ED which illustrates the use of this general inflammatory marker in this particular group of patients.

Of interest is the finding that exacerbations tended to be consistent. If you were eosinophilic at the index event, then you were likely to have a raised blood eosinophil level at subsequent events. This relationship raises the possibility of either there being a common response to the trigger for the AECOPD in some patients. This, in turn, may lead to new therapeutic strategies to treat AECOPD and also perhaps prevent the events occurring.

This study has several limitations. The data collected were retrospective electronic case note review. We were unable to confirm the diagnosis by checking clinical records or through lung function testing, but these events were all associated with ICD-10 criteria. These data are similar to other studies that in a prospective manner have demonstrated associations between mortality and both physiological and biological variable.^23^ We only collected a limited data set, but this was over an 18-month period, which would take into account seasonal variation. The data set was collected at the entry of the patient to the ED. Data on pre-treatment with oral corticosteroids were collected but were thought to be inconsistent and poorly reported. It is possible that pre-treatment may have affected the results but given the size of the data collection, it is likely that any treatment effect would have been matched between those with raised blood eosinophils and those without. These limitations are similar in other studies using similar methodology which has to balance the scale of data collected with the researcher’s ability to check the integrity of the data at the time of entry. It is thus necessary to follow on with a prospective observational study collecting data live with data and diagnostic integrity. Further confirmation of these findings will also be made available through the British Thoracic Society National COPD audit, which is now prospective and continuous, although does not focus on ED attendances. The mortality data should be interpreted with caution as we have not made any corrections for any expected underlying mortality rate driven by the age of the individual patients. However, we believe that the magnitude of this effect is enough to certainly drive future studies as an important endpoint.

## Conclusion

Throughout the world, blood tests are done at the time of presentation to ED in many thousands of patients with AECOPD every day. Our data suggest that routinely collected biochemical markers could be used to prognosticate clinical care, particularly the eosinophil count. These findings must be confirmed in prospective clinical trials.
